# Accelerated Cortical Osteolysis of Metatarsals in Charcot Neuroarthropathy: A Cross‐Sectional Observational Study

**DOI:** 10.1002/jbm4.10243

**Published:** 2019-11-05

**Authors:** David R. Sinacore, Kirk E. Smith, Kathryn L. Bohnert, David J. Gutekunst, Jeffrey E. Johnson, Michael J. Strube

**Affiliations:** ^1^ Department of Physical Therapy High Point University High Point NC USA; ^2^ Department of Biomedical Informatics UAMS Medical Center Little Rock AR USA; ^3^ Program in Physical Therapy Washington University St. Louis MO USA; ^4^ Department of Physical Therapy St. Louis University St. Louis MO USA; ^5^ Department of Orthopedic Surgery, St. Louis School of Medicine Washington University St. Louis MO USA; ^6^ Department of Psychology, St. Louis School of Medicine Washington University St. Louis MO USA

**Keywords:** RADIOLOGY; FRACTURE RISK ASSESSMENT; PRACTICE/POLICY‐RELATED ISSUES; AGING; BIOMECHANICS; ORTHOPEDICS

## Abstract

Metatarsals are frequent sites of stress and fragility fractures in younger athletic populations and aging older adults. Metatarsal fractures are particularly common in Charcot neuroarthropathy (CN), a complication of diabetes mellitus (DM) and peripheral neuropathy (PN). Neuropathic metatarsal fractures may be caused by an accelerated cortical bone osteolysis and may be reflected as geometric‐derived strength estimates from standard foot radiographs. The purpose of this cross‐sectional study was to determine geometry and strength‐derived estimates of the metatarsals in individuals with DM, PN, and CN compared with younger and older adult controls who were nondiabetic and nonneuropathic. We studied 62 participants: 20 young adult controls (YACs), 22 older adult controls (OACs), and 20 diagnosed with DMPN&CN. From weight‐bearing radiographs, we measured the outer diaphysis diameter and inner marrow diameter at the distal, middle, and proximal diaphysis sites of the second and fifth metatarsal. From these diameters, we derived strength estimates of combined cortical width (CCt.Wi), percent cortical area (%Ct.rA), buckling ratio (BR), moment of inertia (MOI), and section modulus (SM) at each site in both metatarsals. DMPN&CN participants had an accelerated cortical thinning, decreased %Ct.Ar, increased BR, and lower MOI and SM compared with OACs and YACs. The OACs showed age‐related decreases in CCt.Wi and % Ct.Ar, and increased BR. The BR demonstrated significant group × bone × site interaction with the distal fifth metatarsal in the DMPN&CN group having the lowest bone strength. The BR in the distal fifth metatarsal of DMPN&CN participants was 36% and 49% greater than in the OAC and YAC groups, respectively. DMPN&CN participants have lower metatarsal bone strength estimates compared with younger and older adult controls. Standard foot radiographs demonstrate an accelerated cortical osteolysis in DMPN&CN individuals, particularly in the distal fifth metatarsal diaphysis. © 2019 The Authors. *JBMR Plus* published by Wiley Periodicals, Inc. on behalf of American Society for Bone and Mineral Research © 2019 The Authors. *JBMR Plus* published by Wiley Periodicals, Inc. on behalf of American Society for Bone and Mineral Research.

## Introduction

Human pedal bones, particularly the metatarsals, are common sites of fracture because of the high loading, bending, and twisting forces that occur during weight‐bearing while walking, running, and jumping, as well as the rapid changes in direction required in activities of daily living and recreation.^1^, ^2^, ^3^ Bone loss (osteolysis) occurs normally as a consequence of advancing age, thereby elevating the risk of metatarsal fractures. However, metatarsal osteolysis is often accelerated in many arthritic, neuropathic, and metabolic conditions such as rheumatoid arthritis or osteoarthritis, spinal cord injury, stroke, osteoporosis, Paget disease, and diabetes mellitus (DM), which may increase the risk of fracture. Studies demonstrating an accelerated osteolysis of metatarsals that accompany aging, neuropathic, and metabolic conditions have heretofore been limited. Most previous studies of bone loss in the human foot have been limited to studies of a single, easily accessible tarsal bone such as the calcaneus using techniques like DXA,^4^ QUS,^5^ or single‐slice CT.^6^ The calcaneus, however, is comprised almost entirely (>80%) of cancellous bone^5^ with a thin cortical shell that envelopes a rich, cancellous microarchitecture. Therefore, any loss of the cortical bone component in the calcaneus can be extremely difficult to detect and quantify by conventional methods, making the calcaneus a poor choice of foot bones to study cortical osteolysis in the human foot.

By contrast, human metatarsals are comprised largely of cortical bone throughout the entire length of the diaphysis; therefore, they may be a more appropriate choice to determine cortical osteolysis that accompanies bone pathologies and their associated risk for metatarsal fracture. Although standard foot radiographs often can reveal diffuse patterns of low bone mass (osteopenia) or osteoporosis in select pedal bones, radiographs are generally not recommended to quantify the magnitude of bone loss in either the cancellous or cortical compartments because of their lack of precision.^7^ Moreover, it is difficult to detect and quantify the extent of cancellous bone loss versus cortical bone loss in human metatarsal bones that is caused by normal aging or DM with peripheral neuropathy‐ (PN‐) related disorders.

Despite several studies reporting a rapid pedal osteolysis over short periods resulting in an elevated risk for new or recurrent fracture in Charcot neuroarthropathy (CN),^8^, ^9^, ^10^, ^11^ there have been few studies of radiographically derived methods of bone loss in human metatarsal bones. Therefore, our purpose was to compare radiographic‐derived metatarsal geometry and estimates of metatarsal strength in the second and fifth metatarsals in young and older adult controls to participants with DM, PN, and CN (DMPN&CN). We tested the null hypothesis that metatarsal geometry and derived strength estimates would not be altered in participants with DMPN&CN compared with younger and older adult controls who were nondiabetic, nonneuropathic, and did not have CN. In addition, we aimed to determine the intra‐ and interrater reliability of radiogrammetric measures of the second and fifth metatarsals by novice raters.

Our underlying premise was that cortical osteolysis would manifest as a deterioration in metatarsal diaphysis geometry and strength‐derived indices, which is accelerated by DMPN&CN. We selected the second metatarsal because it represents the longitudinal midline axis of the foot; is commonly the longest, most‐dense metatarsal in the human foot; and is often a site for stress‐related fracture.^1^ We also selected to study the fifth metatarsal because it represents a lateral column metatarsal that is a common site of fracture along its diaphysis.^3^, ^8^ Our study design also allowed us to compare age‐ and DMPN&CN‐induced changes in radiogrammetric‐derived indices of human metatarsal geometry and strength in young adult controls who have presumably attained their peak metatarsal bone content.

## Participants and Methods

### Participants

The study was conducted at Washington University School of Medicine (St. Louis, MO, USA) and was approved by its Human Research Protection Office's Institutional Review Board. Participants were recruited through local advertisements; written informed consent was obtained from each participant. Participants were remunerated for their participation. Briefly, eligibility criteria included: (1) for young adult control (YAC) participants, an age <45 years and without a current or recent 1‐year history of foot or ankle impairments or deformities; (2) for older adult control (OAC) participants, an age ≥55 years without DM or PN or major midfoot and hindfoot deformities; and (3) as the primary index group, participants with an acute or subacute stage of CN, with a diagnosis of DM (type 1 or 2) with Semmes‐Weinstein monofilament‐confirmed evidence of PN.^12^, ^13^ The sample size for each group of control participants was based on matching the number in the primary index group. Each participant was included if they had at least a single unilateral standing anteroposterior (AP) foot radiograph in the past year. Individuals who reported or whose medical records indicated they were treated with bone‐acting drugs (eg, estrogen‐replacement therapy, bisphosphonate, or glucocorticoid compounds) during the previous year were excluded from this analysis.

### Study design

Three groups of participants were studied in a cross‐sectional comparison of radiogrammetric‐derived geometric and strength parameters of the second and fifth metatarsal bones at each of three diaphysis sites (ie, proximal, middle, and distal diaphysis). Standard weight‐bearing dorsoplantar AP radiographs of at least one foot were assessed by raters who were blinded to one another. Radiographs of only the affected foot of the DMPN&CN group were used and values were averaged from radiographs of both feet (when available) in the YAC and OAC groups. All radiographs were uploaded and imported into ISite PACS (Picture Archiving and Communication Systems; Philips Healthcare Informatics, Foster City, CA, USA) software program for measurement. The ISite PACS software measures angles to the nearest 1‐degree increment and distance measures to the nearest 0.1‐mm increment. A calibration ruler was included in each weight‐bearing radiograph to correct for any magnification error.

#### *Intrarater and interrater reliability of metatarsal geometry measurements*

Two novice raters performed all the radiogrammetric measurements of metatarsal geometry. Each novice rater underwent instruction, training, and practice independently of one another. Raters made measures independent of one another and were instructed not to discuss the results with each another.

**Figure 1:**
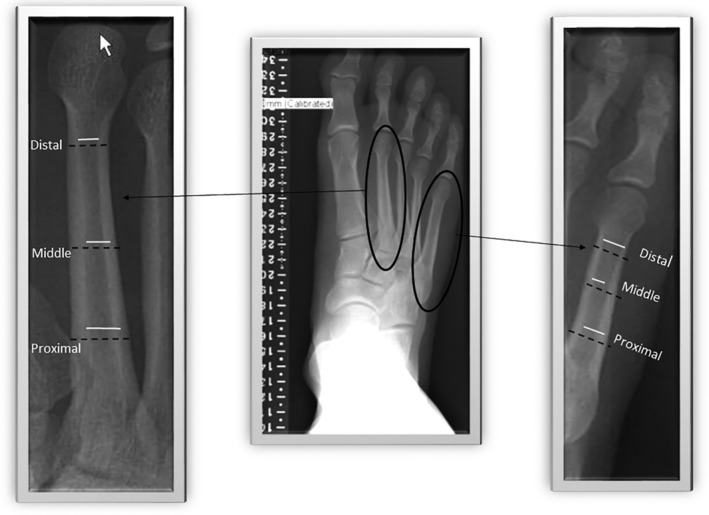
Radiogrammetric measures of outer bone diameter (B.Dm) and inner marrow diameter (Ma.Dm) of the second and fifth metatarsals at the proximal, middle, and distal diaphysis site of each metatarsal. Black dashed lines = B.Dm; white solid lines = Ma.D.

To assess intra‐ and interrater reliability, each rater measured the same 30 radiographs on two separate occasions with a 3‐ to 14‐day interval between repeated measures. Radiographs for intra‐ and interrater reliability were randomly selected from all (right and left feet) available radiographs by one of the authors (KLB) from participant numbers assigned from all three groups of participants included in the study. Both raters were blinded to group allocation of radiographs and blinded to each other's measures throughout the study. Each rater assessed and recorded the length of each metatarsal as the distance between lines drawn tangent through the extreme points of each metatarsal. Each rater determined the proximal (approximately 65% to 75%), middle (50%), and distal (25%) distances from the distal (head) region to make measurements of cortical geometry (Fig. 1). The outer bone diameter (B.Dm in mm) and the inner marrow diameter (Ma.Dm in mm) were assessed directly from each of the foot radiographs.^14^

#### *Metatarsal bone geometry and strength indices*

From the geometry measurements, we calculated cortical geometry and bone strength parameters.^14^, ^15^, ^16^, ^17^ Two variables were calculated using the algorithms described by Meema^14^ and three variables calculated using algorithms described by Beck and colleagues.^16^ We calculated the following parameters: (1) combined cortical width (CCt.Wi; in mm), (2) the percent cortical area (%Ct.Ar); (3) the cross‐sectional areal moment of inertia (MOI; mm^4^); (4) site buckling ratio (BR; unitless), and (5) the section modulus (SM; mm^2^). At the distal, middle, and proximal diaphysis of both metatarsals, we modeled each metatarsal as a circular annulus with 100% of the cortical bone area at a constant radius from the center of the circle to the outer cortical (periosteal) edge. The CCt.Wi was calculated by subtracting the Ma.Dm from the B.Dm at each of the three sites along the diaphysis.^14^ The %Ct.Ar was calculated at each site by the formula: [π · (*B.Dm/*2)^2^ *–* (π *· (Ma.Dm/*2)^2^)/*(*π *·* (*B.Dm/*2)^2^] *× 100*.^14^ The cross‐sectional MOI, BR, and SM were calculated as strength indices of bone quality, assuming each metatarsal was represented as a hollow circular tube with a uniform single wall cortical width similar to what Beck and colleagues had previously done in the hip (ie, proximal femur).^15^ The cross‐section MOI, BR, and SM were geometrical strength indices that corresponded to the metatarsal's resistance to bending and torsion.^11^, ^17^, ^18^ The areal MOI was calculated using the formula, MOI = π/4 * [*r*_*o*_^4^ – *r*_*i*_^4^], where *r*_*o*_ = outer bone radius and *r*_*i*_ = inner marrow radius. The cross‐section MOI was used to calculate the section modulus (mm^2^), calculated as MOI divided by the outer bone radius (*r*_*o*_). The BR for each of the three diaphysis sites was computed as the ratio of the outer bone radius (*r*_*o*_) divided by the average single cortical wall width (½ CCt.Wi).^16^

### Statistical analyses

Participant characteristics were compared using one‐way ANOVAs or Fisher exact tests. We determined the intra‐ and interrater reliability from estimates of intraclass correlation coefficients (ICC) using fixed effects model for each of the two novice raters. The primary dependent variables included in the intra‐ and interrater reliability analysis included the measured variables‐ metatarsal length, B.Dm, and Ma.Dm at each of the three sites on each metatarsal as parameters of metatarsal geometry. Because all bone strength parameters were calculated from the geometric variables, no reliability estimates were required.

We used a 3 × 2 × 3 (group × bone × site) fixed effect analysis of covariance (ANCOVA): with group treated as the between‐subject factor, bone and site treated as the within‐subject factors, and body weight included as a covariate. Group differences were not adjusted for age because age accounted for group variation by design. The ANCOVA group main effect tested our primary null hypothesis of no group differences in metatarsal geometry or derived‐strength estimates. Within the framework of the ANOVA when the *p* value for an effect was significant, follow‐up comparisons were conducted to isolate the nature of the effect. For main effects, all pairwise comparisons were examined. For interactions, we took a simple main effects approach. Pairs of means for one of the variables involved in the interaction were compared within each level of the other variable involved in the interaction (or each combination of levels if the interaction is three‐way). Follow‐up comparisons were adjusted using the Holm procedure to control the type I error rate at .05. Dependent variables tested included B.Dm, Ma.Dm, CCt.Wi, %Ct.Ar, MOI, BR, and SM at the proximal, middle, and distal diaphysis sites of the second and fifth metatarsal bones. When assumption tests occasionally indicated violations of either normality, homogeneity of variance–covariance matrices or sphericity, we conducted randomization tests to verify all inferences. Data are presented in the tables as adjusted mean ± adjusted SD. An alpha level ≤0.05 was significant. All statistical analyses were performed in R version 3.5.1^19^ and R Studio.^20^

## Results

**Figure 2:**
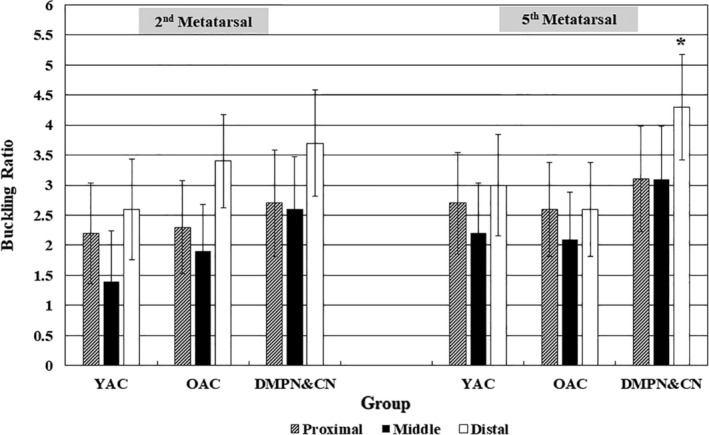
Buckling ratio in young adult control (YAC), older adult control (OAC), and participants with diabetes mellitus, peripheral neuropathy, and Charcot neuroarthropathy (DMPN&CN). Values are adjusted mean ± 95% CI in the proximal, middle, and distal diaphysis site of the second and fifth metatarsals. *p = .056 for group × bone × site (three‐way) interaction.

Participant characteristics are shown in Table 1. The groups did not differ in physical characteristics including sex, race, height, shoe size, and metatarsal length. All groups of participants differed in age (by design); however, participants with DMPN&CN weighed more with a greater BMI compared with YAC and OAC participants. Intra‐ and interrater reliability estimates are shown in Table 2. Intrarater reliability estimates were high for all geometric variables measured, demonstrating excellent reproducibility of radiogrammetric variables in both metatarsals. Tables 3 and 4 and Fig. 2 show the measured and calculated radiogrammetric variables for each group of participants and for the three sites along each of the second and fifth metatarsals, respectively.

**Table 1:** Physical Characteristics of Group Participants

	Young adult control (*n* = 20)	Older adult control (*n* = 22)	DMPN&CN (*n* = 20)	*p* Value
Age (year)	28 ± 6	62 ± 7	55 ± 9	<.05
Female sex, no. (%)	9 (45)	11 (50)	10 (50)	NS
White race, no. (%)	16 (80)	17 (77)	15 (75)	NS
Height (cm)	173 (10)	173 (12)	175 (8)	NS
Weight (kg)	79 ± 18	83 ± 16	113 ± 26	<.05
BMI (kg/m^2^)	26.2 ± 5	27.9 ± 5	37 ± 7	<.05
Shoe size (cm)^a^	42 ± 3	43 ± 4	43 ± 4	NS
2nd metatarsal length (mm)	82 ± 3	82 ± 7	84 ± 8	NS
5fth metatarsal length (mm)	77 ± 7	79 ± 6	77 ± 4.5	NS

Values are means ± SD. DMPN&CN = diabetes mellitus, peripheral neuropathy, and Charcot neuroarthropathy; NS = not significant.

a Shoe size in European expression.

**Table 2:** Intra‐ and Interrater Intraclass Correlation Coefficients for Radiogrammetric Measures of Second and Fifth Metatarsals

	Second metatarsal	Fifth metatarsal
Intrarater		
Metatarsal length	0.995	0.962
Outer bone diameter @ middle	0.998	0.979
Inner marrow diameter @ middle	0.991	0.982
Outer bone diameter @ distal	0.995	0.992
Inner marrow diameter @ distal	0.968	0.976
Outer bone diameter @ proximal	0.989	0.832
Inner marrow diameter @ proximal	0.913	0.811
Interrater		
Metatarsal length	0.968	0.893
Outer bone diameter @ middle	0.990	0.918
Inner marrow diameter @ middle	0.941	0.959
Outer bone diameter @ distal	0.992	0.976
Inner marrow diameter @ distal	0.937	0.956
Outer bone diameter @ proximal	0.986	0.399
Inner marrow diameter @ proximal	0.887	0.474

Values are intraclass correlation coefficients (ICC) for fixed effects model (3, k).

**Table 3:** Radiogrammetric Variables of Second Metatarsal

	Young adult control (*n* = 20)	Old adult control (*n* = 22)	DMPN&CN (*n* = 20)	Statistically significant pairwise differences
Proximal				
Outer bone diameter (mm)	11.0 ± 0.5	11.9 ± 1.0	10.7 ± 1.6	c
Inner marrow diameter (mm)	5.9 ± 0.5	6.6 ± 1.2	6.5 ± 1.6	a ^,^ b
Combined cortical width (mm)	5.1 ± 0.4	5.3 ± 0.8	4.8 ± 1.9	a ^,^ c
% Cortical area	70 ± 3	68 ± 8	64 ± 14	b
Buckling ratio	2.2 ± 0.2	2.3 ± 0.3	2.7 ± 0.8	b ^,^ c
Moment of inertia (mm^4^)	704 ± 132	929 ± 318	617 ± 454	c
Section modulus (mm^2^)	123 ± 18	151 ± 40	108 ± 58	c
Middle				
Outer bone diameter (mm)	8.8 ± 0.4	9.5 ± 0.6	8.7 ± 0.7	c
Inner marrow diameter (mm)	3.5 ± 0.4	4.1 ± 0.8	4.7 ± 1.0	a ^,^ b
Combined cortical width (mm)	5.3 ± 0.5	5.3 ± 0.6	4.0 ± 0.6	b ^,^ c
% Cortical area	84 ± 3	80 ± 5	69 ± 8	a ^,^ b ^,^ c
Buckling ratio	1.6 ± 0.1	1.8 ± 0.1	2.5 ± 0.3	a ^,^ b ^,^ c
Moment of inertia (mm^4^)	309 ± 58	412 ± 116	279 ± 148	c
Section modulus (mm^2^)	68 ± 10	84 ± 17	60 ± 23	c
Distal				
Outer bone diameter (mm)	8.2 ± 0.7	8.4 ± 0.9	7.8 ± 0.8	c
Inner marrow diameter (mm)	5.1 ± 0.7	5.6 ± 1.6	5.4 ± 1.6	a ^,^ b
Combined cortical width (mm)	3.1 ± 0.3	2.8 ± 0.6	2.4 ± 0.5	a ^,^ b ^,^ c
% Cortical area	61 ± 7	56 ± 14	51 ± 13	a ^,^ b ^,^ c
Buckling ratio	2.8 ± 0.5	3.3 ± 1.3	3.6 ± 1.7	a ^,^ b ^,^ c
Moment of inertia (mm^4^)	195 ± 31	205 ± 44	147 ± 40	c
Section modulus (mm^2^)	46 ± 6	47 ± 7	36 ± 6	c

Values are adjusted means ± SD after adjustment for body weight. Pairwise comparisons based on significant group main effects with Holm procedure adjustment to protect type I error rate. DMPN&CN = diabetes mellitus, peripheral neuropathy, and Charcot neuroarthropathy; YAC = young adult control; OAC = older adult control.

a YAC versus OAC.

b YAC versus DMPN&CN.

c OAC versus DMPN&CN.

**Table 4:** Radiogrammetic Variables of Fifth Metatarsal

	Young adult control (*n* = 20)	Old adult control (*n* = 22)	DMPN & CN (*n* = 20)	Statistically significant pairwise differences
Proximal				
Outer bone diameter (mm)	11.5 ± 1.3	11.8 ± 0.8	10.4 ± 1.2	b ^,^ c
Inner marrow diameter (mm)	6.0 ± 0.5	6.7 ± 1.0	6.2 ± 1.5	a ^,^ b
Combined cortical width (mm)	5.1 ± 0.7	4.5 ± 1.0	3.7 ± 1.6	a ^,^ c
% Cortical area	70 ± 3	60 ± 11	55 ± 14	a ^,^ c
Buckling ratio	2.2 ± 0.2	2.9 ± 1.0	3.2 ± 1.2	b ^,^ c
Moment of inertia (mm^4^)	873 ± 598	815 ± 258	654 ± 394	c
Section modulus (mm^2^)	142 ± 65	135 ± 33	118 ± 51	c
Middle				
Outer bone diameter (mm)	9.0 ± 0.6	9.1 ± 0.8	8.2 ± 0.6	b ^,^ c
Inner marrow diameter (mm)	3.5 ± 0.4	4.2 ± 0.7	4.7 ± 0.9	a ^,^ b
Combined cortical width (mm)	4.8 ± 0.7	4.3 ± 1.0	3.7 ± 1.6	b ^,^ c
% Cortical area	77 ± 5	70 ± 10	58 ± 16	b ^,^ c
Buckling ratio	1.9 ± 0.2	2.2 ± 0.6	3.2 ± 1.4	b ^,^ c
Moment of inertia (mm^4^)	327 ± 98	345 ± 152	259 ± 120	c
Section modulus (mm^2^)	70 ± 16	71 ± 23	59 ± 19	c
Distal				
Outer bone diameter (mm)	8.1 ± 0.6	8.1 ± 0.8	7.2 ± 0.7	b ^,^ c
Inner marrow diameter (mm)	5.1 ± 0.7	5.6 ± 1.4	5.5 ± 0.9	a ^,^ b
Combine cortical width (mm)	3.5 ± 0.4	2.8 ± 0.4	1.8 ± 0.5	a ^,^ b ^,^ c
% Cortical area	67 ± 6	57 ± 8	43 ± 12	b ^,^ c
Buckling ratio	2.3 ± 0.3	2.9 ± 0.6	4.5 ± 1.5	a ^,^ b ^,^ c
Moment of inertia (mm^4^)	198 ± 86	194 ± 132	143 ± 87	c
Section modulus (mm^2^)	47 ± 12	45 ± 16	39 ± 9	c

Values are adjusted means ± SD after adjustment for body weight. Pairwise comparisons based on significant Group main effects with Holm procedure adjustment to protect type I error rate. DMPN&CN = diabetes mellitus, peripheral neuropathy, and Charcot neuroarthropathy; YAC = young adult control; OAC = older adult control.

a YAC versus OAC.

b YAC versus DMPN&CN.

c OAC versus DMPN&CN.

### Group differences

All groups differed significantly in geometric and strength‐derived estimates (Tables 3 and 4). Pairwise comparisons for B.Dm indicated the YACs and OACs are not different from each other, though the DMPN&CN group was different from both control groups. For Ma.Dm, pairwise comparisons indicated the YAC group was different from the OAC and DMPN&CN groups, but OAC and DMPN&CN groups were not different. Pairwise comparisons for CCt.Wi indicated the DMPN&CN group differed from the YAC and OAC groups, but the control groups were not different. BR and %Ct.Ar were different between all groups. MOI and SM in the DMPN&CN group were significantly lower than YAC group, but not the OAC group; the YAC and OAC groups were not different from each other.

### Bone differences

There were differences in geometric and strength‐derived estimates between the metatarsals with the fifth metatarsal having lower CCt.Wi and %Ct.Ar and higher Ma.Dm and BR compared with the second metatarsal. MOI and SM were not different between the second or fifth metatarsals.

### Diaphysis site differences

Geometric and strength‐derived estimates were different at all diaphysis sites. For MOI, B.Dm, and SM, the distal site was lower compared with the middle site, which in turn was different from the proximal sites. For CCt.Wi, the middle and proximal sites were lower than the distal site, but not different from each other. For %Ct.Ar, the distal site was lower than the middle site, which was lower than the proximal site. For BR, the middle site was lower than the proximal site, which was lower than the distal site. For Ma.Dm, the middle site was lower than the distal site, which was lower than the proximal site.

### Group × bone interaction

There was a group × bone interaction for CCt.Wi, %Ct.Ar, and BR. Interactions were evaluated in two ways: First, bone differences were tested within each group, and then group differences were tested for each bone. For CCt.Wi, there were differences between the second and fifth metatarsals in the OAC and DMPN&CN groups, but not the YAC group. The second approach indicated group differences for the second metatarsal; the DMPN&CN group was different from the YAC and OAC groups, which were not different from each other. For the fifth metatarsal, all groups differed from one another. For %Ct.Ar, there were significant bone differences for the DMPN&CN and OAC groups, but not the YAC group. Also, for the second metatarsal, the DMPN&CN group was different from the YAC, but not the OAC group. The control groups were not different from one another. For the fifth metatarsal, all groups were different from one another. For BR, there were bone differences for the DMPN&CN group, but not for either control group. For group differences tested for each bone, the second metatarsal in the DMPN&CN group was different from the YAC group, but not different from the OAC group. The control groups were not different from one another. In the fifth metatarsal, all groups were different from one another. There were no group × bone interactions for B.Dm, Ma.Dm, %Ct.Ar, MOI, or SM.

### Group × site interactions

There were group × site interactions for Ma.Dm and BR. These interactions were expressed in two ways: Sites were tested within each group, and then group differences were tested for each site. The first approach for Ma.Dm showed that all diaphysis sites were different from each other in the YAC and OAC groups. In the DMPN&CN group, the Ma.Dm at the proximal site was different from the middle and distal sites, which were not different from each other. The second approach showed that for the Ma.Dm distal site, none of the groups was different, but the middle site in the YAC group was different from the OAC and DMPN&CN groups, which were not different from each other. For Ma.Dm at the proximal site, the OAC group was different from the YAC group, but not from the DMPN&CN group. The DMPN&CN group was not different from the YAC group. Using the first approach of testing site differences within each group, we found that for the BR all sites were different from all other sites for both control groups. For the DMPN&CN group, the distal site was different from the middle and proximal sites, which were not different from each other. Using the second approach of testing for group differences at each site, the BRs in all groups were different from each other for the distal site. For the middle site, the DMPN&CN group was different from the YAC and OAC groups, which were not different from each other. For the proximal site, DMPN&CN group was different from the YAC group, but not different from the OAC group. There were no differences between the control groups. There were no group x site interactions for B.Dm, CCt.Wi, %Ct.Ar, MOI, or SM.

### Bone × site interactions

There were bone × site interactions for B.Dm, Ma.Dm, CCT.WI, %Ct.Ar, and BR, but not for MOI or SM. These interactions were expressed in two ways: first, testing for site differences within each bone, and then testing for bone differences at each site. For B.Dm, we found significant bone differences at the distal site, and for both the second and fifth metatarsals, each site was different from one another. For Ma.Dm, there were significant bone differences for the middle and proximal sites, but not for the distal site; for the second metatarsal, each site was different from each other. For the fifth metatarsal, middle and distal sites were different from the proximal site, but not different from each other. For CCt.Wi, the first approach found there were bone differences for the middle and proximal sites, but not for the distal site. The second approach found that for the second metatarsal, the distal site was different from the middle and proximal sites, which were not different from each other, whereas for the fifth metatarsal, all sites were different from each other. For %Ct.Ar and BR, the first approach showed there were bone differences for the middle and proximal sites, but not for the distal site. For the second and fifth metatarsal, each site was different from each of the other sites.

### Group × bone × site interactions

BR is the only variable that demonstrated a marginally significant (*p* = .06) group × bone × site interaction, where the DMPN&CN's distal site BR on the fifth metatarsal averaged 4.5 (highest), resulting in the lowest resistance to fracture (Fig. 2). There were no other significant three‐way interactions for any of the other geometric or strength‐derived estimates.

## Discussion

This is the first study to report radiogrammetric measures of metatarsal geometry and derived strength indices caused by aging and DMPN&CN. The major finding was that in DMPN&CN participant's feet, the distal and proximal diaphysis CCt.Wi and %Ct.Ar were significantly decreased (Tables 3 and 4), resulting in a dramatic increase in BR (Fig. 2) at those sites and a greatly diminished resistance to bending, twisting, and loading moments, which markedly increased the risk for further stress‐related or neuropathic fragility fracture.^8^ The observed decreases in CCt.Wi and %Ct.Ar with resultant increases in BR of both metatarsals exceeded the adaptations in the OAC group and can be attributed to a combination of DM, distal polyneuropathy, and CN (characterized by a persistent inflammatory osteolysis).^9^, ^10^ BRs in the distal fifth metatarsal of DMPN&CN participants were 36% and 49% greater than than OAC and YAC groups, respectively (Table 4, Fig. 2).

A further finding was that cortical osteolysis of the second and fifth metatarsal in DMPN&CN participants did not appear to be uniform between metatarsals nor throughout the diaphysis sites compared with the OAC group. The fifth metatarsal had lower CCt.Wi and %Ct.Ar, and higher BR at all diaphysis sites compared with the second metatarsal, which was exacerbated in the DMPN&CN group. A greater endosteal expansion may have occurred in the distal and proximal diaphysis or perhaps there was greater periosteal apposition in the middle diaphysis in the feet of the DMPN&CN group compared with aging adults. These findings suggest a single middle diaphysis site in a single metatarsal may not be the best in vivo location for cortical osteolysis assessment or the optimal location as a predictor of stress‐ or neuropathic fragility fracture.

The second major finding of our study was that both aging and DMPN&CN were associated with a homeostatic expansion (increase) in the outer (periosteal) and inner (marrow) diaphysis diameters in both the second and fifth metatarsals. The periosteal expansion in B.Dm represents a well‐known homeostatic compensatory adaptation in the aging of long bones that compensates for the age‐related osteolysis at the cortical envelope, but maintains the areal cross‐sectional MOI and SM and attempts to preserve the resistance to fracture.^21^, ^22^ Apposition of new bone on the periosteal surface redistributed the cortical bone radially at the periphery and around the geometric center of the metatarsal diaphysis. The periosteal expansion maintained the bone's structural strength despite an obligatory cortical osteolysis as evidenced by the diminishing %Ct.Ar and CCt.Wi with an increasing BR.^21^ The BR was increased because of the concurrent expansion of the outer and inner diaphysis diameters, resulting in a thinning of the cortical wall with aging. The impact on the resistance to bending moments was maintained with only a slightly elevated BR (small decrease in resistance to bending moments) despite the age‐associated cortical osteolysis.

Additionally, this is the first study to report the intra‐ and interrater reliability estimates of radiographic‐derived indices of metatarsal geometry by novice raters. The intra‐ and interrater ICC reliability estimates were high for all sites along the second metatarsal and distal and middle diaphysis sites in the fifth metatarsal. The exception to high reliability estimates was the proximal end (base) in the fifth metatarsal, where interrater ICC estimates were poor (ie, 0.39 to 0.47). The likely explanation for the poor interrater ICC estimates at the proximal base of the fifth metatarsal is that there was much overlap with the fourth metatarsal base overlapping with the fifth metatarsal base on the AP radiograph, therefore obscuring the outer and inner bone diameters on the radiographs. Each rater clearly measured their same proximal region of the fifth metatarsal at time 1 and again at time 2 as demonstrated by high intrarater ICC values; however, the proximal site may have differed markedly in location between each rater accounting for poor interrater agreement at the proximal site.

There have been previous studies of human metacarpals^23^, ^24^ and metatarsals^25^ using radiogrammetry to demonstrate cortical osteolysis in rheumatoid arthritis and after hallux valgus correction, respectively, but our study represents the first study of cortical osteolysis of metatarsals in participants with DMPN&CN. Our findings confirm the results of several studies, namely that aging results in the loss of cortical bone (eg, a reduced CCt.Wi and %Ct.Ar) that affects metatarsal strength estimates.^26^ Long bones adapt by periosteal apposition and endosteal expansion with only a small increase in the BR at all sites, with a resultant corresponding small increase in susceptibility to metatarsal fracture with aging.

The utility of assessing geometric strength indices of the metatarsals, particularly the BR and SM, has been previously verified. Gutekunst and colleagues demonstrated that BR and SM at the middle site of the second and third metatarsal calculated from volumetric quantitative CT scans accounted for 89% of the variance in ex vivo failure loads using three‐point mechanical bending. BR and SM as geometric strength indices, in combination with density‐weighted BMD, were highly predictive of ex vivo metatarsal failure loads.^27^ Therefore, geometric strength indices may hold promise as in vivo predictors of future metatarsal fracture using standard digital X‐ray radiogrammetry. We believe these image‐derived strength indices can be used to predict the onset of metatarsal stress‐related or neuropathic fragility fracture.^28^ Although a definitive fracture threshold for each human metatarsal has yet to be identified, we have observed that as the BR approaches or exceeds a value of ≥3, particularly in the proximal and distal diaphysis regions of the fifth metatarsal, it may signal a decreasing (lower) resistance to bending and twisting forces and may signal an increased risk for stress‐related or fragility‐induced metatarsal fracture.^28^

The results from our radiogrammetric study of human metatarsal cortical bone loss are consistent in magnitude with what we have observed using quantitative ultrasonometry in the calcaneus in subjects with acute CN.^9^ Previously, we reported an 18% loss of bone density (mixed cortical and cancellous bone) in the involved foot of subjects with an acute CN compared with healthy, age‐matched control subjects.^9^ Although the calcaneus was nearly 80% to 90% cancellous bone, these radiogrammetric methods in the metatarsals confirmed 5% to 7% cortical area osteolysis had occurred in DMPN&CN participants who were younger than the OAC participants at the distal diaphysis site, and a 15% to 18% loss in cortical area in DMPN&CN participants at the distal diaphysis site compared with YAC participants in both second and fifth metatarsals. As cortical osteolysis accelerates, BR increases, making human metatarsals susceptible to fracture and unable to resist bending stresses associated with normal activities compared with younger adults. As BR approaches or exceeds a value of 3 or greater,^28^ the risk for metatarsal fracture increases as resistance to bending moments decrease.

There are limitations to our study. Foremost, the design of our current study did not allow us to discern the distinct contributions of disease (diabetes), neuropathy, or acute CN on metatarsal osteolysis. To directly address the distinct contributions of each, additional control groups including non‐Charcot neuropathic and nonneuropathic participants will need to be included. Our previous work suggests that an acute or prolonged inflammation associated with CN is largely the incipient contributor of the 5% to 6% cortical osteolysis in the metatarsals^10^ because participants with DM and PN, but without CN, gained an average of 0.4% apparent BMD after 1‐year follow‐up.^10^ Also previously, participants with DMPN and a neuropathic plantar ulcer recovered QUS‐derived calcaneal BMD 1 year after immobilization, whereas participants with DMPN&CN did not recover calcaneal BMD after immobilization: This implies that diabetes and neuropathy are not the primary contributing factors in pedal bone loss and recovery.^29^

Another limitation is when estimating the metatarsal strength indices, we assumed the metatarsals to be uniformly circular with a constant diameter (and radius). Similarly, we assumed the cortical width (and area) around the marrow cavity was uniform and therefore can be represented by a single average cortical width. Although these assumptions allowed us to quickly derive and estimate the structural indices as described by Beck and colleagues for increasing fragility of the hip,^16^ they may in fact either over‐ or underestimate the diameters (and radii) of the metatarsals if they are more oval‐shaped or irregularly circular. Similarly, if disease or stresses on the metatarsals affect one cortical wall of the metatarsal (ie, a single cortical width), then we may slightly over‐ or underestimate a given site's strength index. Another potential limitation using digital X‐ray radiogrammetry is the difficulty in defining the outer and inner diameters in cases where there are focal periosteal (edge) defects present or irregularly shaped marrow cavity edges that are nonuniformly expanding. In the present study, this challenge occurred primarily in the proximal fifth metatarsal diaphyseal site in the DMPN&CN participants and less frequently in the YAC and OAC participants. Periosteal edge defects (eg, holes or nonmineralized callus) reduce the true mineralized cortical thickness and decrease the cortical area with a resultant increase in the BR (ie, increasing the risk of metatarsal fracture). Despite these challenges, digital X‐ray radiogrammetry is a fast, highly reliable and informative method to assess the impact of cortical osteolysis of the metatarsals that occurs in individuals with metabolic and arthritic bone diseases.^14^, ^24^

In summary, our results show an accelerated cortical osteolysis in feet of individuals with DM, PN, and CN compared with older adults. These changes arise from *increases* in diaphysis diameters with *decreases* in cortical geometry (combined or single wall width and area), resulting in significant *increases* in BR at the distal, middle, and proximal diaphysis of the second and fifth metatarsals in participants with DMPN&CN. In comparison, smaller adaptations in cross‐sectional MOI and SM at the metatarsal shafts were observed in the DMPN&CN and OAC groups to maintain resistance to bending, despite structural adaptations that accompanies aging. Cortical osteolysis and an increasing BR are easily identified and assessed using standard digital foot X‐ray radiogrammetry. An increasing BR may signal an increasing risk for stress‐related and fragility‐induced metatarsal fractures.

## Disclosures

All authors state that they have no conflicts of interest.
